# Reduced Resting Metabolic Rate in Adults with Hemiparetic Chronic Stroke

**DOI:** 10.4172/2155-9562.1000341

**Published:** 2015-12-22

**Authors:** Monica C Serra, Charlene E Hafer-Macko, Alice S Ryan

**Affiliations:** 1 Department of Veterans Affairs, Baltimore VAMC, Division of Gerontology and Geriatric Medicine, Department of Medicine, University of Maryland School of Medicine, Baltimore, USA; 2 Department of Veterans Affairs, Baltimore VAMC, Department of Neurology, University of Maryland School of Medicine, Baltimore, USA

**Keywords:** Resting metabolic rate, Chronic stroke, Weight management

## Abstract

**Objective:**

Resting metabolic rate (RMR) is the component of energy expenditure that explains the largest proportion of total daily energy requirements. Since RMR is determined largely by fat-free mass and a low RMR predicts weight gain in healthy adults, identifying the role of muscle atrophy following stroke on RMR may help identify ways to mitigate the development of obesity post-stroke.

**Methods:**

Thirty-nine stroke survivors with chronic hemiparesis (mean ± SEM: age: 61 ± 1 years, latency from stroke: 107 ± 40 months, BMI: 31 ± 3 kg/m2) underwent DXA scans for measurement of body composition, including total, paretic, and non-paretic leg lean mass and fasted, 30-min indirect calorimetry for measurement of RMR.

**Result:**

Predicted RMR was calculated by the Mifflin-St Jeor equation, which considers weight, height, and age for both men and women. RMR was 14% lower than predicted (1438 ± 45 vs. 1669 ± 38 kcals/24 hrs; P<0.01). Total (r=0.73, P<0.01), paretic (r=0.72, P<0.01) and non-paretic (r=0.67, P<0.01) leg lean mass predicted RMR.

**Conclusion:**

These data indicate that muscle atrophy post stroke may lead to a reduced RMR. This substantiates the need to attenuate the loss of lean mass after a stroke to prevent declines in RMR and possible weight gain common post-stroke.

## Introduction

Stroke is the leading cause of long-term disability [1]. We have previously shown that resultant hemiparesis leads to lean tissue wasting and decreased strength [2,3], which may impair and delay post-stroke recovery. The decline in muscle mass and strength following stroke are directly related to increased frailty, dependency, disability and falls [4–6]. Moreover, loss of muscle may contribute to declines in energy expenditure [7] and the subsequent weight gain [8] observed post-stroke. Thus, determining optimal strategies to maintain energy expenditure and energy balance (expenditure=intake) could be important to offset potential weight gain after stroke.

Total daily energy expenditure (TDEE) consists of resting metabolic rate (RMR), the thermic effect of food, and physical activity. Resting metabolic rate (RMR) is the component of energy expenditure that explains the largest proportion of total daily energy requirements. Individuals with a low RMR are at higher risk of significant weight gain, relative to those with a high RMR [9,10]. Although the effect of acute stroke on hypermetabolism has been examined [11–14], presently, only one study has examined RMR in chronic (>6 months latency) stroke [15]. de Sant’Anna [15] found that RMR of stroke survivors with hemiparesis is ~two fold higher than that of age and BMI matched non-stroke adults; however, several limitations of this study (i.e. accuracy of methods used to assess body composition and RMR) affect its clinical interpretability. Declines in fat mass, independent of changes in lean mass, do not appear to result in a decrease in RMR [16]; however, loss of muscle mass observed with other (non-stroke) chronic diseases [17], aging [18], prolonged bed rest [19], and detraining [20] are associated with a reductions in RMR. It is suggested that changes in muscle mass of 4.5 lbs can change RMR by ~50 kcals/day [21]. Since it is well established that RMR is determined largely by fat-free mass, accounting for ~60–70% of RMR [22] and large proportion of total body lean mass is found in the extremities, we hypothesis that muscle atrophy of the lower extremity may contribute to a reduced RMR post-stroke. Thus, the aim of this study was to determine RMR in chronic stroke and whether leg lean mass predicts a reduced RMR.

## Methods

This cross-sectional study included 39 stroke survivors, between the ages of 45–80 years, which were recruited from the Baltimore area for participation in exercise rehabilitation studies. Participants were in the chronic phase of stroke recovery (at least six months prior) and had residual hemiparetic gait deficits. All volunteers signed University of Maryland Institutional Review Board approved informed consent forms.

Participants underwent a health history and physical examination, which included height, weight, blood pressure, and a resting electrocardiogram. Dual-energy X-ray absorptiometry (DXA) scans (iDXA; Lunar Radiation, Madison, WI) were conducted to determine total body fat (%) and total, paretic, and non-paretic leg lean tissue mass.

Subjects received two weeks of heart healthy diet (<30% of calories from total fat, <10% of calories from saturated fat) counseled by a Registered Dietitian prior to RMR testing. Subjects reported to our lab first thing in the morning following a 12 hour fast. RMR was measured by indirect calorimetry (COSMED; Rome, Italy), while participants rested quietly in the supine position under a ventilated canopy for 30 minutes in a thermo-neutral and comfortable environment. Energy expenditure was calculated by the Weir equation [23] and expressed per 24 hours. Predictive RMR was calculated using the Mifflin-St Jeor equation [24], which considers weight, height, and age for both men and women. We selected the Mifflin-St Jeor equation since this newer equation is more likely to predict measured RMR than other methods [25].

Descriptive statistics were analyzed using SPSS (PASW Statistics, Version 18, Chicago, IL). Results are expressed as mean ± SEM. Student’s t-tests were used to compare predicted to measured RMR. Linear regression was used to assess whether leg lean mass predicts RMR. Statistical significance was set at a two-tailed P < 0.05.

## Results

**Figure 1:**
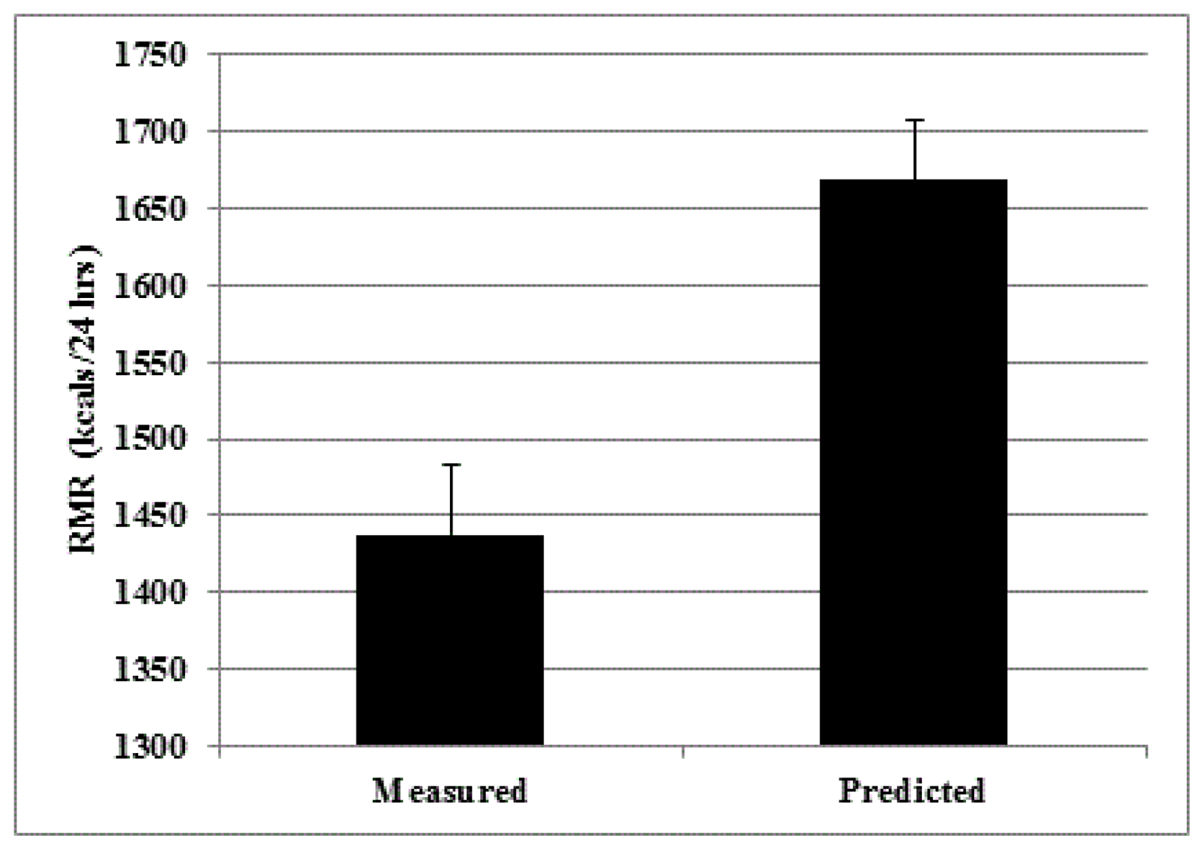
Resting metabolic rate (measured by indirect calorimetry) is 14% below predicted (using Mifflin-St Jeor equation) in chronic stroke survivors with hemiparesis (P<0.01).

Subject characteristics may be viewed in Table 1. Subjects were 72% male, 49% African American, 46% Caucasian, and 5% Hispanic. On average, subjects were ~60 years old, obese, and ~9 years post-stroke. When compared to predicted values, RMR was 14% lower (P<0.01; Figure 1).

**Figure 2:**
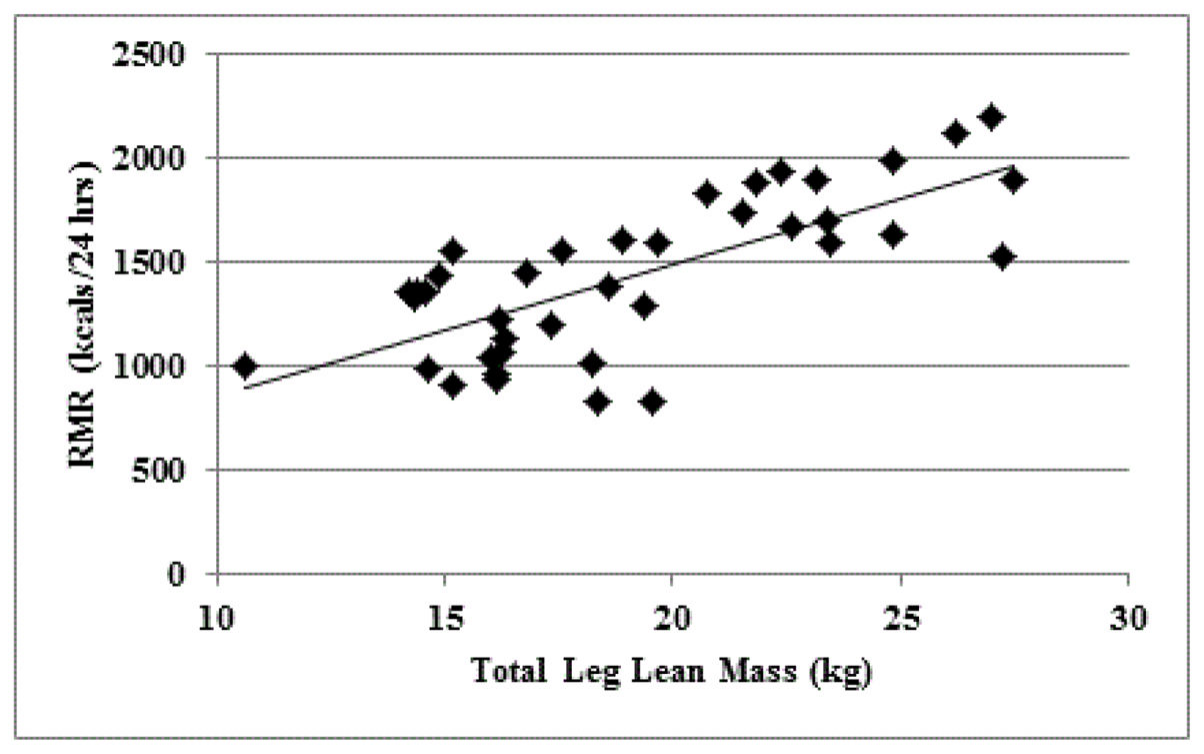
Total leg lean predicts RMR in stroke survivors with hemiparesis (r=0.73, P<0.01).

RMR was not associated with younger age (r=−0.12, P=0.46) or latency from stroke (r=−0.07, P=0.83). However, we observed that total body (r=0.70) and total leg (r=0.73, Figure 2) lean tissue mass predicted RMR (P’s<0.01). This also was true when each leg individually was used to predict RMR (paretic: r=0.72 and non-paretic: r=0.67; P’s<0.01).

## Discussion

Chronic hemiparesis is present in nearly 50% of all long-term ischemic stroke survivors [1]. In addition to reductions in energy expenditure due to physical inactivity common with prolonged hemiparesis, we hypothesized that hemiparesis also may affect energy balance through stroke-induced muscle atrophy. Our data indicate that muscle atrophy post stroke may lead to a reduced RMR as we observed that RMR during the chronic phase of stroke is significantly lower than predicted. However, our data also point to the need to maintain overall lower extremity lean mass since it is a strong predictor of RMR regardless of paretic leg muscle atrophy following a stroke with chronic hemiparesis.

Our observation that RMR is lower than predicted is relevant to stroke recovery since studies suggest that lower RMR is a predictor of greater sequential weight change [26]. Further, maintenance of weight loss or weight gain is associated with compensatory changes in resting energy expenditure [27]. The low RMR post-stroke may further contribute to energy imbalance caused by physical inactivity. Our findings contrast the one previously published report of RMR in chronic stroke where RMR was ~two fold higher in stroke survivors compared to non-stroke controls [15]. Several differences in methodology may explain these variances. de Sant’Anna et al. [15] only required four hours of fasting in their measurement of RMR; yet, evidence suggests that 12 hours of fasting is required to ensure dietary nutrient clearance from the circulation [28]. RMR was only measured for 20 minutes in this previous study, while it is suggested that the accuracy of RMR is maximized if at least 30 consecutive minutes are measured [29]. Further, subjects were required to breathe through a mouthpiece with a nasal clip attached, which may have caused discomfort, preventing complete relaxation. We believe that our 30 min measurement of RMR using a ventilated hood, following 12 hours of fasting, may better indicate resting values.

Despite the small sample size, this study is strengthened by the inclusion of a diverse study population, including a wide range of ages (middle aged to elderly), ethnicities, and body composition (normal to morbidly obese). Since the incidence of stroke is higher in men than women (aged less than 75 years) [30], we believe this indicates that our predominately male gender distribution is representative of the general stroke population. Similar differences in lean mass between the paretic and non-paretic leg using DXA are observed previously by our group in chronic stroke [2,3] and others in acute stroke [31], further indicating generalizability to other stroke populations. We previously showed that lean tissue differences between the paretic and non-paretic legs are greater using computed tomography (CT) versus DXA scans [2]. It is possible that the relationship between RMR and lean mass could differ between legs if using a more sensitive measure of body composition, such as CT scans. Identifying a difference in the relationship between paretic versus non-paretic leg lean mass and RMR would further highlight muscle atrophy as an important target to prevent RMR changes and subsequent weight gain post-stroke.

Research efforts for effective treatment strategies to maintain a healthy body weight focus on diet and exercise programs. While we have previously reported improvements in body composition (muscle hypertrophy and decreased fat mass) following the initiation of exercise during the chronic phase of stroke [32–34], how these interventions affect RMR has yet to be reported. Less is known regarding dietary intake and how it could influence RMR post-stroke. This is despite evidence that albumin concentrations are lower in chronic stroke compared to non-stroke controls [35], indicating that protein intake is inadequate, which could lead to loss of muscle tissue. In non-stroke populations, protein supplementation can promote muscle protein synthesis, especially when combined with exercise training [36,37]. Future studies could examine the role of muscle hypertrophy on RMR and weight management following these lifestyle interventions during the chronic phase of stroke.

In summary, we observed that stroke survivors are at risk for a low RMR in the chronic phase of stroke. It appears that preserving overall lower extremity muscle mass may contribute in the maintenance of RMR after a stroke. This substantiates the need to attenuate the loss of lean mass, possibly with lifestyle interventions, after a stroke to prevent declines in RMR and possible weight gain common post-stroke.

## Figures and Tables

**Table 1:** Subject characteristics

	Mean ± SEM	Range
Age (yrs)	62 ± 1	46 – 75
Stroke Latency (mo)	107 ± 40	14 – 423
Weight (kg)	92 ± 3	55 – 134
BMI (kg/m2)	31 ± 1	19 – 45
Total Body Fat (%)	36.8 ± 1.3	10.7 – 52.5
Total Body Lean Mass (kg)	55.9 ± 1.7	33.7 – 82.0
Total Leg Lean Mass (kg)	19.1 ± 0.7	10.6 – 27.4
Paretic Leg Lean Mass (kg)	9.2 ± 0.4	5.0 – 13.8
Non-Paretic Leg Lean Mass (kg)	9.8 ± 0.4	2.9 – 14.4
RMR (kcals/24 hrs)	1,438 ± 45	928 – 2,204

## References

[1] American Heart Association I (2013). Older Americans & Cardiovascular Diseases. Statistical Fact Sheet.

[2] Ryan AS, Dobrovolny CL, Smith GV, Silver KH, Macko RF (2002). Hemiparetic muscle atrophy and increased intramuscular fat in stroke patients. Arch Phys Med Rehabil.

[3] Ryan AS, Buscemi A, Forrester L, Hafer-Macko CE, Ivey FM (2011). Atrophy and intramuscular fat in specific muscles of the thigh: associated weakness and hyperinsulinemia in stroke survivors. Neurorehabilitation and neural repair.

[4] Tsur A, Segal Z (2010). Falls in stroke patients: risk factors and risk management. Isr Med Assoc J.

[5] Kim O, Kim JH (2015). Falls and Use of Assistive Devices in Stroke Patients with Hemiparesis: Association with Balance Ability and Fall Efficacy. Rehabil Nurs.

[6] Patten C, Lexell J, Brown HE (2004). Weakness and strength training in persons with poststroke hemiplegia: rationale, method, and efficacy. J Rehabil Res Dev.

[7] Michael KM, Allen JK, Macko RF (2005). Reduced ambulatory activity after stroke: the role of balance, gait, and cardiovascular fitness. Arch Phys Med Rehabil.

[8] English C, Thoirs K, Coates A, Ryan A, Bernhardt J (2012). Changes in fat mass in stroke survivors: a systematic review. Int J Stroke.

[9] Ravussin E (1995). Low resting metabolic rate as a risk factor for weight gain: role of the sympathetic nervous system. International journal of obesity and related metabolic disorders: journal of the International Association for the Study of Obesity.

[10] Buscemi S, Verga S, Caimi G, Cerasola G (2005). Low relative resting metabolic rate and body weight gain in adult Caucasian Italians. Int J Obes (Lond).

[11] Frankenfield DC, Ashcraft CM (2012). Description and prediction of resting metabolic rate after stroke and traumatic brain injury. Nutrition.

[12] Finestone HM, Greene-Finestone LS, Foley NC, Woodbury MG (2003). Measuring longitudinally the metabolic demands of stroke patients: resting energy expenditure is not elevated. Stroke.

[13] Esper DH, Coplin WM, Carhuapoma JR (2006). Energy expenditure in patients with nontraumatic intracranial hemorrhage. JPEN J Parenter Enteral Nutr.

[14] Bardutzky J, Georgiadis D, Kollmar R, Schwarz S, Schwab S (2004). Energy demand in patients with stroke who are sedated and receiving mechanical ventilation. J Neurosurg.

[15] de Sant’Anna M, Eboli LC, Silva JG, Dos Santos AG, Lourenco M (2014). Resting metabolic rate analysis in chronic hemiparesis patients. Neurol Int.

[16] Connolly J, Romano T, Patruno M (1999). Selections from current literature: effects of dieting and exercise on resting metabolic rate and implications for weight management. Fam Pract.

[17] Schrack JA, Knuth ND, Simonsick EM, Ferrucci L (2014). “IDEAL” aging is associated with lower resting metabolic rate: the Baltimore Longitudinal Study of Aging. J Am Geriatr Soc.

[18] Holliday MA, Potter D, Jarrah A, Bearg S (1967). The relation of metabolic rate to body weight and organ size. Pediatr Res.

[19] Molé PA (1990). Impact of energy intake and exercise on resting metabolic rate. Sports Med.

[20] Ormsbee MJ, Arciero PJ (2012). Detraining increases body fat and weight and decreases VO2peak and metabolic rate. J Strength Cond Res.

[21] Donnelly JE, Jakicic JM, Pronk NP, Smith BK, Kirk EP (2003). Is resistance training effective for weight management: A review of the evidence. Evidence Based Preventive Medicine.

[22] Johnstone AM, Murison SD, Duncan JS, Rance KA, Speakman JR (2005). Factors influencing variation in basal metabolic rate include fat-free mass, fat mass, age, and circulating thyroxine but not sex, circulating leptin, or triiodothyronine. Am J Clin Nutr.

[23] Cunningham JJ (1990). Calculation of energy expenditure from indirect calorimetry: assessment of the Weir equation. Nutrition.

[24] Mifflin MD, St Jeor ST, Hill LA, Scott BJ, Daugherty SA (1990). A new predictive equation for resting energy expenditure in healthy individuals. Am J Clin Nutr.

[25] Frankenfield D, Roth-Yousey L, Compher C (2005). Comparison of predictive equations for resting metabolic rate in healthy nonobese and obese adults: a systematic review. J Am Diet Assoc.

[26] Ravussin E, Lillioja S, Knowler WC, Christin L, Freymond D (1988). Reduced rate of energy expenditure as a risk factor for body-weight gain. N Engl J Med.

[27] Leibel RL, Rosenbaum M, Hirsch J (1995). Changes in energy expenditure resulting from altered body weight. N Engl J Med.

[28] Cox RA, Garcia-Palmieri MR (1990). Cholesterol, Triglycerides, and Associated Lipoproteins. Clinical Methods: The History, Physical, and Laboratory Examinations.

[29] Smyrnios NA, Curley FJ, Shaker KG (1997). Accuracy of 30-minute indirect calorimetry studies in predicting 24-hour energy expenditure in mechanically ventilated, critically ill patients. JPEN J Parenter Enteral Nutr.

[30] Mozaffarian D, Benjamin EJ, Go AS, Arnett DK, Blaha MJ (2015). Heart disease and stroke statistics--2015 update: a report from the American Heart Association. Circulation.

[31] Iversen E, Hassager C, Christiansen C (1989). The effect of hemiplegia on bone mass and soft tissue body composition. Acta Neurol Scand.

[32] Ivey FM, Ryan AS, Hafer-Macko CE, Goldberg AP, Macko RF (2007). Treadmill aerobic training improves glucose tolerance and indices of insulin sensitivity in disabled stroke survivors: a preliminary report. Stroke.

[33] Ryan AS, Ivey FM, Prior S, Li G, Hafer-Macko C (2011). Skeletal muscle hypertrophy and muscle myostatin reduction after resistive training in stroke survivors. Stroke.

[34] Ivey FM, Ryan AS (2014). Resistive training improves insulin sensitivity after stroke. J Stroke Cerebrovasc Dis.

[35] Serra MC, Hafer-Macko CE, Ivey FM, Macko RF, Ryan AS (2014). Impact of serum nutritional status on physical function in african american and caucasian stroke survivors. Stroke research and treatment.

[36] Rahbek SK, Farup J, Moller AB, Vendelbo MH, Holm L (2014). Effects of divergent resistance exercise contraction mode and dietary supplementation type on anabolic signalling, muscle protein synthesis and muscle hypertrophy. Amino Acids.

[37] Paoli A, Pacelli QF, Neri M, Toniolo L, Cancellara P (2015). Protein supplementation increases postexercise plasma myostatin concentration after 8 weeks of resistance training in young physically active subjects. J Med Food.

